# Beyond Borders: Exploring the Experiences and Challenges of Ghanaian Nurses in Barbados Through Video-Based Analysis

**DOI:** 10.1155/jonm/8401519

**Published:** 2025-05-26

**Authors:** Emmanuel Graham Nyameke, John Windie Ansah, Isaac Defiin

**Affiliations:** Department of Sociology and Anthropology, University of Cape Coast, Cape Coast, Ghana

**Keywords:** bilateral agreement, Ghanaian nurses, migration, nurse experiences, nurse migration

## Abstract

Migration of nurses continues to be a problem facing most African countries because governments are not able to absorb graduate nurses into the local health system. Because of this, some governments have resorted to bilateral agreements with other western countries to export nurses. Ghana has not been immunised against this social phenomenon. The government, through a bilateral agreement with the government of Barbados, has sent some nurses to the island. However, among the studies that have looked at the experiences of migrant nurses, the challenges and experiences of Ghanaian nurses in Barbados have not been explored. This paper explored the experiences and challenges of Ghanaian nurses in Barbados using video-based analysis, relying on three already existing videos of some of the nurses on YouTube. It was found that although the political environment was not favourable, the nurses were happy with the government's decision to export nurses. Further, it was found that the government of Ghana does not play its supervisory role over the contracts to ensure that items in the contract are observed, which affects the nurses sent there.

## 1. Introduction

The discourse on nurse migration has attracted scholars from all walks of life (Abuosi and Abor [1]; Boafo [2]; Adhikari and Melia [3] and Anarfi et al. [4]). However, a few studies have paid attention to the export of nurses by some countries. The development of technology has facilitated easy access to information, which has facilitated nurse migration. While the migration of nurses from developing countries is profitable for some western countries, some African countries have ceased the export of nurses through bilateral agreements as a way of dealing with their unemployment situation and also as a means of revenue mobilisation. In Ghana, because of the inability of the government to absorb all trained nurses into the local health sector, the government chose the export of nurses as a way to deal with the situation. In a quest to deal with the situation, the government has chosen to export experienced nurses to make way for other young nurses to be recruited into the local health sector. In 2019, upon a request from the Barbados Prime Minister, the government of Ghana sent 95 special nurses to Barbados in the year 2020 [5]. Further, the Vice President of the Republic of Ghana in 2022 noted that the country will be sending additional nurses to Barbados [6]. Confirming that Ghanaian nurses had arrived in Barbados, Forde, a news reporter for Barbados Today, reported in 2022 that an additional 122 specialised Ghanaian nurses had arrived to work on the Island.

According to the desk information given to the researchers from the ministry of health, the Barbados contract was to last for 2 years but subject to renewal. The in charge of policy had disclosed that the nurses who have been exported have the liberty to renew their contract as agreed upon. Unlike nurses who migrate on their own and do not have bilateral arrangements, in the case of nurses exported by the government, they are regulated through some bilateral arrangement or under a memorandum of understanding. Such arrangements spell out the form the contract takes, how issues are to be regulated, how disputes are settled, and as specified how workers will benefit from the contract under the international labour migration regulations [7]. It has been more than 3 years since the first cohort of nurses were sent there; however, the government has not done much to investigate the experiences of the nurses sent to Barbados in order to inform future policy on exporting nurses. The objective of this paper is to explore the experiences and challenges of Ghanaian nurses in Barbados.

## 2. Literature Review

Nurses who emigrate to other countries in search of employment, as well as nurses sent on missions or exported by their countries, face a variety of difficulties [8]. Foreign nurses have faced many challenges while working abroad. These include cultural barriers, language barriers and different healthcare systems. Over the years, nurses have gained different lived experience across the countries they have worked in.

### 2.1. Language Barrier

Language barriers can also be difficult to manage, as nurses may not have a complete understanding of communications with their patients and colleagues. The authors of [9], in their study on migrant nurses from the Philippines working in Norway, noted that those nurses faced communication barriers. Migrant nurses found it difficult to communicate with patience and with their colleagues. Kishi et al. [10]; Nichols et al. [11]; Taylor [12]; Walsh and O'Shea [13] and Xu et al. [14], in their respective studies, have noted that communication barriers have been one major issue some migrant nurses face. Migrant nurses who find themselves in countries that speak languages other than their own face communication issues. Communicating with patients and other hospital staff becomes difficult. Ellahham [15] also observed in his study that there are situations where migrant nurses speak the language of the destination country, but there may be different accents, which may become a barrier to communication. In a study by Bonvillain [16], it was found that language was one of the most important tools for helping nurses adapt to their new surroundings, which was a difficult task. This was a problem for migrant nurses. Because of this, when migrants are unable to communicate, it affects their productivity.

### 2.2. Adaptation Issues

Also, nurses who move to other countries to work have trouble adjusting to their new surroundings. Accordingly, Nortvedt et al. [9] conducted a study and revealed that nurse migrants who found themselves in a new environment had difficulty integrating into that environment because they could not speak the language. Another difficulty migrant nurses experience is cultural barriers. Literature accounts for the fact that cultural barriers can be difficult to overcome, as nurses must understand the values and beliefs of their patients in order to provide appropriate care [17].

### 2.3. Skill Transition Issues

Generally, migrant nurses face transition problems. Nurse migrants go through difficulties in adopting the practices of their profession in an environment that is new to them. Literature says that migrant nurses face transition problems [18]. Migrant nurses who get used to how things are done in their home countries face a challenge when they try to do things the way they used to do them in their home hospitals in the new hospitals they find themselves in. Also, some migrant nurses end up working in remote villages and home care facilities, which does not help them learn and improve their skills [3].

### 2.4. Discrimination Issues

Walani [19] acknowledged in his study that migrant nurses who had their studies outside their countries and are working in those countries face some discrimination at work. Nurses with foreign educations who work in the countries where they studied face job discrimination. Their colleagues see them as outsiders.

Blythe et al. [20] also account for the fact that over the years, nurses who moved to other countries have had trouble with regulations when they got there. Nurse migrants are faced with immigration challenges upon arrival. These challenges leave them frustrated. There is the problem of getting licenced in the destination before practicing.

Nurses who work in overseas hospitals face cultural shocks. Nurses are faced with new cultural values, some of which are different from their old culture. They have to go through the situation of unlearning their old culture to learn the new culture, which usually comes with some challenges [14]. This literature falls short of the new investigation since the unit of study was Asians working in the US, who have entirely different cultures compared to Africans working in the US and UK and may have different experiences.

African nurses working abroad have also gathered some experiences from working abroad. Likupe [21] conducted a study into the experiences of African nurses working in the UK and noted that they experienced racism and discrimination and were also not given equal opportunities.

### 2.5. Source of Enriching Skills

Nursing professionals who work abroad often report an enriching and exciting experience. For many, it is an opportunity to gain global insight through healthcare systems, engage in a different culture, learn a new language and build professional networks. According to the 2019 AACN Global Nursing Survey, ‘most respondents reported a variety of positive experiences while working internationally'. These included a greater appreciation of cultural diversity, a heightened understanding of other cultures and improved confidence in working in different healthcare systems [22].

For some, building their skills and gaining invaluable international experience make up for the challenges that come with working abroad. Cultural, language and healthcare system differences all present unique challenges, in addition to being far from family support. The reward of the experience, however, is worth the effort. According to one nurse working in the Middle East, You are very well taken care of in the healthcare organisations. You get to learn different ways of caring for patients, use new and updated technology and resources and meet new people. You even end up making very close friends. ‘It's an unforgettable experience' [23]. The varied lived experiences of nurses abroad demonstrate their adaptability and resilience in undertaking this challenging yet rewarding career opportunity. Those willing to take on the challenge have the potential to reap the benefits of learning new skills, collaborating and immersing themselves in a different culture.

In conclusion, although the above literature has explored some of the experiences of migrant nurses, the literature on the experiences of migrant nurses who are sent on government programmes has not been very much explored.

## 3. Aim of the Study

This essay's main goal was to explore the experiences of Ghanaian nurses who worked in Barbados on behalf of the government of Ghana.

### 3.1. Methodology

A YouTube video-based analysis was conducted using three different YouTube videos in which the leaders of Ghanaian nurses who have been sent to work in Barbados by the government of Ghana shared some of their experiences. It was okay for the researcher to make use of existing video data for research since the use of these video data provided issues that were key for video-based research. Hence, the researcher used three already existing video datasets. Accordingly, Walker and Boyer [24] quoting [25, 26] acknowledged that existing videos, documentaries and films could be used as sources of data for research. Swain and King [27] also observed that outside the formal interview with participants, if the participant continues to speak and the researcher realises that the information is still valuable to the research, the researcher can ask for permission to record or take notes and use them in the research. From the above literature, it was possible for the researcher to use an already existing video recording where some participants shared some important information on the research being conducted. It is against this background of established methods that the use of YouTube videos as a source of data is justified. On data search, the authors inputted into the Google browser ‘videos of Ghanaian nurses in Barbados' these three video links dropped. Upon initial scrutiny of the videos to find out if they met what the researcher was looking for, those videos were considered. To be sure of the nurses in the video being among the nurses exported to Barbados, the faces in the video were matched with faces in a picture taken at the Barbados international airport when the nurses landed and were being received by the Barbadian government. Two participants were involved in one of the videos (those were the leaders of the first cohort of nurses sent there), and one of the nurses who appeared in the same video with another nurse appeared in two different videos alone. In all, there were two participants for the study. The links to these videos have been included in this work. https://www.youtube.com/watch?v=yr9C6DeOJEk, https://www.youtube.com/watch?v=rP5xGCs-s14 and https://www.youtube.com/watch?v=hHPzlDLWihA.

Thematic narrative analysis and interpretive phenomenological analysis were employed to analyse the data. The thematic analysis was used to help identify imaging themes after the data were coded manually. Identifying the emerging themes made the authors identify similar issues from the participants. After the identification of the themes, in analysing the data, although it was based on themes, the specific issues under the themes were best analysed and interpreted using the IPA. The IPA helped to delve deeper into the findings as they were analysed under themes to help bring out the lived experiences of the nurses in Barbados. In order to process the data for analysis, the researcher downloaded the data, listened to the videos and transcribed the data by listening, pausing and typing after the data have been typed into word document, the others proofread the transcripts to and also made sure the transcript had no or a few grammatical errors. Later, which the transcribed data were coded manually and the researchers developed themes as Fiaveh [28] postulates. The researchers used a prose writing style in presenting the results and the discussion. The use of this presentation style allowed the researcher to always pause in the middle of the discussion to comment on participants' views, continue after the commentary and add perspectives from the literature. The prose writing style also allowed the researcher to present the results and discussion in a lively and engaging manner.

### 3.2. Ethical Consideration

This study is a part of a master's thesis and so all ethical issues regarding research were adhered to. To protect the identity of the participants, no real name was used. Further, the researchers got ethical clearance from the Institutional Review Board (IRB) of the University of Cape Coast (UCCIRB/CHLS/2023/46) and the Ghana Health Service Ethical Committee (GHS-ERC 048/05/23).

### 3.3. Limitations

The study has some limitations in terms of the number of participants that were involved. Although 95 nurses had been exported, only two of them had shared their experiences which may not cover the entire experiences of other nurses. Hence, the findings of this study cannot be generalised.

## 4. Results and Discussion

In this section, the researcher discussed the experiences and challenges faced by Ghanaian nurses in Barbados. Migrant nurses have gained some experience over the years. Some of their experiences stretch from hospitality issues to working conditions, language barriers and the lack of supervision on the part of the Ghana government.

### 4.1. Hospitality Situation

Hospitality has been considered a factor that may enable a visitor to continue to stay or want to leave. In the case of the Ghanaian nurses in Barbados, identifying their skin colour with the Bajans made them feel more than welcome. Hospitality has been a major factor in motivating migrants to stay in their new environment, whether local or international. Although what is hospitable may differ from person to person, one of them is the kind of environment you find yourself in, whether the people are welcoming or not, and whether the environment is adaptable and something the migrant can easily relate to. From the transcribed data from the videos, some of the Ghanaian nurses who were sent to work for the Barbados government observed that the environment of Barbados was just as that of Ghana with not much difference, which made them feel like they were in their home country working. Yaw observed,…*There is no difference between Barbadians and us. When you wake up and step out of your room, you don't see any differences. We are all the same. It is only when a Barbadian speaks that you find some differences.* In a similar direction, Yaa also observed,…*I was thinking that I would see Barbados like the cities I see when watching movies, I was shocked. Barbados was just like Ghana.* From the first statement from Yaw, it shows some commonalities between the environment of Ghana and that of Barbados. This meant that the Ghanaian nurses who were sent there to work did not find themselves in a completely new environment. It meant that they felt welcomed because they were all black [29]. Thus, Brown [29] observed in the history of the Bajans that they are mostly black people. Unlike Likupe [21], whose study of African nurses working in the UK was discriminated against because of their colour, Ghanaian nurses working in Barbados did not face racial problems. They rather felt like they were still in Ghana working and were willing to stay and work. This is contrary to Nortvedt et al. [9] who observed in their study that migrant nurses who found themselves in new environments faced integration problems.

In addition, contrary to a general welcoming environment, Ghanaian nurses experienced a politically hostile environment. The nurses faced opposition from the political opposition party in Barbados. The opposition party was not happy with the government for bringing in foreign nurses, and Yaw expressed this in this way,…*In point of fact, the opposition party was pleased with the fact that we were in the country. There were some of those who did not make us feel welcome. But there were those people on the opposing side who made things difficult for us; those who are opposed to the government gave us a hard time, and they continue to make things difficult for us.* Observing a similar experience, Yaa also noted,…*Actually, not all political people were happy we were in the country. Some of them did not welcome us,* and *those against the government gave us a tough time, and they still give us a tough time.* The observations from the two participants were that the opposition party, which is against the government, and like any opposition party will do, opposed the decision made by the government to recruit foreign nurses. And from the statements from Yaw and Yaa, opposition from the opposing political party made things a little difficult for them.

Further, the Ghanaian nurses faced opposition from some of the ordinary people. The people took to social media to express their unhappiness with the presence of Ghanaian nurses in their country. And Yaw remarked that *they were not happy with us being in their country initially, and they expressed it on social media because they say there are neighbouring countries with excess nurses; why go to Africa to recruit nurses? But the story has changed,* he continued*. Through our hard work, we have surprised them, and they are happy to have us now.* Stating why the Bajans initially saw them as a threat, Yaa said this: *they think they have friends and families in the neighbouring countries who could be recruited, but the government recruited people far from their continent. One of the Barbados nurses said to me in a conversation that the Grenadians have excess nurses that they could recruit from, but they went as far as Ghana to recruit.* The Bajans initially were not happy with the nurses from Ghana because they have friends and relatives in the surrounding countries who could be employed, so why did their government have to go all the way to Africa, particularly Ghana, to recruit nurses? Although people were not happy with Ghanaian nurses at the initial stage, the story changed based on the hard work of those Ghanaian nurses. The people have come to love them now.

### 4.2. Language

Further, migrant nurses usually face language barriers and also communication problems. Unlike the observations by various literature such as Kishi et al. [10]; Nichols et al. [11]; Taylor [12]; Walsh and O'Shea [13] and Xu et al. [14], the data from the Ghanaian nurses in Barbados tell a different story. Yaw remarked that… *language was not a problem. We all spoke English. The only problem I face is hearing them when they speak. You need to pay close attention in order to distinguish between the two of them because their accents are really distinctive. S*imilarly, Yaa also said*, Actually, language was not a problem*…*the only problem I face is with hearing them. Their accents are very different, and you have to pay rapt attention to hear them. But they heard me when I spoke.* From the just quoted voices, it was clear that language was not the problem, and that Ghanaian nurses working in Barbados could speak the language of the people because both countries speak English. However, their problem was with the accent of the people, which made it a little difficult for them to hear them when they speak. This is confirmed by Ellahham [15] in his study that there are situations where migrant nurses speak the same language of the destination country, but there may be some difference with the accents and it may become a barrier to communication.

### 4.3. Working Conditions

Further on the experiences and challenges, Ghanaian nurses in Barbados face the challenge of not getting assistance from their colleague workers at work. They observed that unlike working in Ghana, where a nurse gets assistance from colleague nurses when working on a patient, it is different in Barbados. Yaw, one of the Ghanaian nurses observed,…*you know something, these people do not call on you to assist them, so it is difficult to get them to assist you if you need them. They are not prepared to assist you in any way at this time. They will not assist you in any way when you are working on something and require assistance. And even when they are working alone and are in need of assistance, they will not contact anyone to assist them.* From the voice, it means that the Bajan nurses do things independently and will not call on their colleague nurse even if that nurse is a Bajan to come and assist them, unlike Ghanaian nurses, who sometimes look up to getting assistance from their colleague nurses. The Ghanaian nurse who is used to relying on the assistance of colleague nurses to work sometimes finds that offensive when they call on a Bajan nurse colleague for help and they refuse.

### 4.4. Cultural Shock

As part of the experiences of some of the Ghanaian nurses in Barbados was cultural shock. The Ghanaian nurses were shocked to find out that many of the people in Barbados prefer to stay in concubinage than get married. Further, many of the Bajans live in cohabitation. This was a bigger shock to them, as back home in Ghana, they have experienced many of the people getting married rather than staying in concubinage. In Ghana, unlike what they have experienced in Barbados, many of the people prefer marriage to cohabitation or anything else. Describing how shocked that was for Yaw, he said, *most Barbadians are not married. Someone could be dating for 20 or even 30 years with children and be accepted here. I have a colleague who has been dating for 30 years and has four children but is not married. I asked her why they are not married, and she said that they love it that way, and a lot of them want it that way.* Perhaps wanting to test the waters, he wanted to get one of them as a wife, and he refused. Also, it is a popular thing in Ghana that women between the ages of 25 and 30 do everything to get married, but that is different for Barbados. Yaw disclosed his shock as he further revealed that *I even told one of them that I wanted to marry her, and she said no. They are simply not interested in marrying, but they are okay with dating.* To a young Ghanaian man who has travelled abroad and was thinking he could get a woman to marry but was only going to be accepted to date was a big shock for him. While Yaw was expressing this shock, Yaa nodded in agreement.

Also, thinking that Barbadians will have traditional leaders because they have African roots and the fact that they are also black, Yaw was further shocked. He observed this in the following words: …*Barbadians do not have traditional leaders like we have in Ghana. They don't have kings and queens, and they are surprised Ghana has them. They trace their lineage to Africa and even Ghana.* Speaking about traditional leadership, which can be found in most African countries, Yaw discovered this in a conversion with some Bajans and realised that they did not have any traditional leadership. In fact, the mention of kings and queens to them by Yaw was a shock as well to the people he engaged in the conversation.

### 4.5. Nonobservance of Contract Items

Nurse exports or healthcare professional exchanges have operated within some bilateral arrangements between the sending and receiving countries. Through those arrangements, the two countries draft their agreement, which explains what the contract will be like and specifies items in the contract. In the case of the contract that was made between the Ghanaian government and the Barbados government, some of the Ghanaian nurses who have been sent to work in Barbados have observed that some items in the contract are not being observed by the Barbados government. In the interview section, Yaa nodded in agreement as Yaw said, *Oh yes, some of the items in the contract are not practiced on the ground; we are not paid any allowances.* This drives home the message that there are no government agents or individuals responsible to oversee the observance of the contract which means that depending on the number of years the nurses are to work there, they will not get any allowance while their Bajan colleague nurses get. And again, Yaw could not name other items that were not being observed because he was on air, as he noted: *I cannot mention some of them since I am speaking on air*. This means that there are other items in the contract that are not being observed by the Barbados government. This means that the government of Ghana is not playing its supervisory role in ensuring that the items in the contract are observed and that Ghanaian nurses enjoy every benefit they have to enjoy as spelled out in the contract.

### 4.6. Living Conditions

Describing the living conditions in Barbados, the two Ghanaian nurses described it as high. Yaa, the leader of the first group of nurses sent to Barbados had this to say, *living in Barbados is very expensive. Things are expensive here.* The participant acknowledges that the cost of living in Barbados is extremely high. Although the cost of living is very high, she remarked that *despite that I am able to save 30% of my salary and I am able to send my family some money.* From the statement, the narrator sends the message that the cost of living in Barbados is quite expensive compared to it is in Ghana; however, because the salary is good, they are able to save something more than what they could have done if they were in Ghana. Again, this voice from one of the nurses confirms the new economic labour of migration theory that says that migrants will remit part of their monies to households and further confirms Cebolla-Boado et al. [30] that people will migrate to countries where the salaries are better.

## 5. Summary

The researchers sought to explore the experiences and challenges of Ghanaian nurses in Barbados. It was found that the government of Ghana, who has gone into agreement with the government of Barbados, has not put measures in place to ensure that the contract is faithfully observed by the Barbados government, which has affected nurses. They are not getting allowances, although their Bajan colleagues are given allowances, while allowances have been captured in the contract. Also, despite the unfavourable political environment, the Ghanaian nurses are happy with the government's initiative to export nurses.

## 6. Conclusion

Although the nurses in Barbados experienced different forms of life as well as some limitations in the contract, thus no supervision of the execution of the items in the contract on the part of the Ghanaian government, for the government to continue to export the services of nurses and as well reap it benefits, there is the need for the government of Ghana to monitor the contract and see to it that their nurses are well treated and benefit from the fullness of the contract if the government wants to attract more nurses to accept to be exported. There is the need to conduct further research on the exported nurses to bring out their lived experiences in Barbados. Doing so will inform policymakers' plans and measures to be put in place in order for both nurses and the government to benefit from the export of nurses.

## Data Availability

The data that support the findings of this study are available on request from the corresponding author. The data are not publicly available due to privacy or ethical restrictions.
